# New Advances in Bioactive Compounds in Health and Disease: From Molecular Mechanisms to Translational Perspectives

**DOI:** 10.3390/ijms27209165

**Published:** 2026-10-16

**Authors:** Luís R. Silva

**Affiliations:** 1 School of Health, Polytechnic University of Viseu, 3504-510 Viseu, Portugal; luisfarmacognosia@gmail.com; 2 RISE-Health, Faculty of Health Sciences, University of Beira Interior, Av. Infante D. Henrique, 6200-506 Covilhã, Portugal

## 1. Introduction

Bioactive compounds are increasingly positioned at the interface between natural-product chemistry, food science, molecular pharmacology, and translational medicine. Their relevance no longer rests solely on the observation that a plant extract, food-derived molecule, peptide, protein, or natural-product derivative displays antioxidant or anti-inflammatory activity. The central scientific question has shifted toward establishing causal links between chemical identity, biologically achievable exposure, molecular target engagement, pathway modulation, and measurable effects in physiologically relevant systems. This shift is essential if bioactive-compound research is to progress from descriptive bioactivity toward mechanism-based health applications and, where appropriate, therapeutic development ([1,2,3,4], Contributions 1–7).

This evolution is particularly important because bioactive compounds often exhibit pleiotropic and context-dependent effects. A single molecule may influence redox homeostasis, inflammatory signaling, metabolic regulation, gene expression, microbial ecology, or cell differentiation, while complex extracts may contain multiple constituents whose effects are additive, synergistic, antagonistic, or dependent on biotransformation. Consequently, chemical composition, dose, matrix, metabolism, tissue exposure, and host phenotype are integral components of biological interpretation rather than secondary experimental considerations. Recent literature has emphasized that poor bioavailability, structural instability, incomplete mechanistic characterization, and limited predictive validity of conventional preclinical models remain major barriers to clinical translation [1]. Interindividual differences in the absorption, distribution, metabolism, and excretion of phenolic compounds further complicate this landscape, with gut microbiota, genetic polymorphisms, age, sex, body composition, and physiological status contributing to variable exposure and response [2].

The Special Issue “New Advances in Bioactive Compounds in Health and Disease” reflects this changing scientific landscape. Its seven contributions encompass phenolic acids and flavonoids, combinations of plant-derived compounds, a traditional herbal formulation, a food-derived protease inhibitor, and a rationally designed butyrate derivative [Contributions 1–7]. The studies address metabolic dysfunction, inflammation, bacterial and viral processes, and skin biology through approaches ranging from literature synthesis and chromatographic characterization to cell-based assays, molecular docking, transcriptomics, and animal experimentation. Read together, these papers provide more than a catalog of bioactivities: they illustrate different stages of a translational continuum extending from source and chemical characterization to molecular mechanism, biological response, and potential health application.

## 2. Contributions to the Special Issue: From Chemical Diversity to Mechanistic Evidence

Nunes et al. examined phenolic acids recovered from fruit cultivation and processing by-products as potential agents relevant to metabolic syndrome [Contribution 1]. This topic is scientifically significant at two interconnected levels. First, peels, seeds, pomace, leaves, and stems represent chemically rich matrices that can support circular-bioeconomy strategies by converting underused biomass into sources of value-added compounds. Second, phenolic acids such as caffeic, chlorogenic, ferulic, and *p*-coumaric acids have been reported to modulate biological processes associated with metabolic dysfunction. The reviewed evidence links these compounds with antioxidant, anti-inflammatory, antihyperglycemic, antihypertensive, and anti-obesity effects [Contribution 1]. However, the translational value of such observations depends on moving beyond total phenolic content and generic antioxidant assays toward compound-resolved characterization, bioaccessibility, metabolite profiling, pharmacokinetics, and exposure–response relationships. This is particularly relevant because the chemical composition of by-products can vary with genotype, cultivation conditions, maturity, processing, extraction procedure, and storage.

The review by Almatroodi and Rahmani focuses on caffeic acid and illustrates the transition from a chemically defined phenolic compound to a multi-target bioactive candidate [Contribution 2]. Caffeic acid has been associated with modulation of oxidative stress, inflammatory signaling, lipid peroxidation, apoptosis, angiogenesis, cell-cycle regulation, immune responses, and pathways including PI3K/Akt. Preclinical studies support neuroprotective, cardioprotective, hepatoprotective, antimicrobial, anti-obesity, and anticancer activities [Contribution 2]. Yet this breadth of activity also raises an important mechanistic issue: multi-target effects should not be interpreted as evidence of clinical efficacy unless target engagement occurs at concentrations that are realistically attainable in vivo. Poor solubility, rapid metabolism, limited systemic exposure, and incomplete human evidence therefore remain central limitations. In this context, the distinction between the activity of the parent compound and that of circulating or tissue metabolites becomes particularly important.

Rahmani et al. similarly reviewed hesperidin, a citrus flavanone with reported antioxidant, anti-inflammatory, anticancer, hepatoprotective, neuroprotective, nephroprotective, cardioprotective, antimicrobial, and immunomodulatory effects [Contribution 3]. The study also highlights two increasingly relevant research directions: combinations with other bioactive or therapeutic agents and advanced formulation strategies designed to improve exposure. Hesperidin exemplifies a common paradox in natural-product research: extensive biological activity in experimental systems combined with physicochemical and pharmacokinetic properties that restrict translation. Low aqueous solubility, limited intestinal permeability, metabolism, and rapid degradation may substantially modify the relationship between administered dose and biologically active concentration [Contribution 3]. Formulation science and nanodelivery may help address these constraints, but improved delivery must be accompanied by rigorous characterization of pharmacokinetics, biodistribution, safety, and whether enhanced exposure improves the intended biological outcome [1].

The contribution by Tadtong et al. on curcumin and evodiamine addresses another important dimension: the biological consequences of combining bioactive molecules [Contribution 4]. In LPS-stimulated RAW264.7 macrophages, curcumin and the curcumin–evodiamine combination modulated inflammatory mediators, while molecular docking investigated interactions with TNF-*α*, IL-1*β*, iNOS, and COX-2 and frontier molecular orbital calculations provided complementary chemical information [Contribution 4]. The value of this type of integrated design lies in its ability to generate mechanistic hypotheses across experimental scales. Nevertheless, claims of synergy require particularly careful interpretation. A combination may appear more active because of differences in effective concentration, potency, pharmacodynamics, or assay behavior. Future studies should therefore combine formal interaction models with concentration–response matrices, orthogonal target-validation methods, and in vivo exposure data before synergistic therapeutic effects are inferred.

A second study by Tadtong et al. integrates traditional knowledge with modern analytical and computational approaches in a Thai anti-abscess herbal formulation containing *Curcuma zedoaria*, *Vitex trifolia*, and *Azadirachta indica* [Contribution 5]. Validated HPLC–PDA analysis identified curcumin, demethoxycurcumin, bisdemethoxycurcumin, and vitexicarpin among the principal constituents. The formulation showed antioxidant activity and selective antibacterial activity against *Staphylococcus epidermidis*, while docking suggested a possible interaction between curcumin and the teicoplanin-associated transcriptional regulator TcaR [Contribution 5]. Scientifically, this study illustrates a useful progression for complex botanical preparations: phytochemical definition, quantitative standardization, functional testing, mechanistic hypothesis generation. The next translational steps should include direct biofilm assays, confirmation of target engagement, evaluation in physiologically relevant infection models, and determination of whether the concentrations required for antibacterial effects can be achieved safely at the intended site of action.

Wu et al. expand the scope of the Special Issue beyond low-molecular-weight phytochemicals by investigating soybean trypsin inhibitor (SBTI) as a modulator of host factors associated with SARS-CoV-2 infection [Contribution 6]. The authors reported reduced expression of ACE2, TMPRSS2, and CD147 in experimental systems and observed no apparent histopathological alterations in lung, liver, or kidney tissue after 14 days of SBTI administration in mice [Contribution 6]. This study is conceptually important because it shows how food-derived proteins may interact with host pathways relevant to disease. At the same time, modulation of host-associated entry factors should be distinguished from demonstration of antiviral efficacy. Direct viral challenge models, pharmacokinetic and tissue-exposure studies, mechanistic dissection of receptor regulation, longer-term safety assessment, and ultimately human studies would be required to establish clinical relevance.

Swindell et al. provide a different perspective by studying zinc dibutyroyllysinate (ZDL), a novel compound integrating zinc, lysine, and butyrate-related chemical features [Contribution 7]. In a full-thickness human skin model, RNA sequencing revealed broad transcriptional effects, including increased expression of genes related to epidermal differentiation and retinol metabolism and reduced expression of MITF and other melanogenesis-associated genes. Additional experiments indicated effects on collagen I, collagen IV, prolidase, and melanin secretion [Contribution 7]. This contribution demonstrates the value of transcriptomic profiling for moving beyond single-endpoint bioactivity assays toward pathway-level characterization. It also illustrates how rational derivatization can generate bioactive molecules with biological profiles distinct from those of their individual components. The proposed involvement of epigenetic mechanisms, including histone acetylation, is biologically plausible but remains a hypothesis that should be tested directly through chromatin- and target-specific experiments.

Despite their chemical diversity, the seven contributions converge on several mechanistic themes. Oxidative stress and inflammatory signaling remain recurrent biological nodes, but the collection also extends to metabolic control, apoptosis and angiogenesis, bacterial biofilm regulation, host factors implicated in viral entry, epidermal differentiation, extracellular-matrix biology, and pigmentation [Contributions 1–7]. This diversity reinforces an important concept: bioactive compounds should increasingly be investigated within molecular networks rather than through isolated endpoints. Complex diseases are rarely governed by a single pathway, and the apparent advantage of many natural compounds may lie in their capacity to modulate interconnected targets. The challenge is to distinguish biologically coherent multi-target activity from nonspecific reactivity.

A useful framework emerging from the Special Issue is therefore: (i) source and chemical identity, (ii) standardized preparation, (iii) bioaccessibility and metabolism, (iv) molecular target engagement, (v) pathway response, (vi) cellular and tissue phenotype, (vii) organism-level effect, and (viii) human validation. Weakness at any step can compromise interpretation of the next. For example, a strong in vitro effect has limited translational meaning when the active concentration cannot be achieved in plasma or target tissue; a docking result is hypothesis-generating rather than evidence of direct target binding; and changes in biomarkers do not necessarily establish a clinically meaningful health effect. Conversely, integrating orthogonal evidence across these levels can substantially strengthen causal inference.

The increasing use of multi-omics approaches offers an important opportunity to achieve this integration. Genomics, transcriptomics, proteomics, and metabolomics can support natural-product discovery, target identification, pathway mapping, and biomarker development, while high-resolution analytical platforms can link biological responses to defined chemical entities and metabolites [3]. The transcriptomic strategy used for ZDL in this Special Issue [Contribution 7] exemplifies this shift toward systems-level analysis. Future work should increasingly combine chemical profiling with multi-omics readouts and functional validation, rather than treating compositional analysis and biological testing as separate research streams.

Computational methods also have an expanding role, particularly in target prioritization, molecular docking, network pharmacology, structure–activity analysis, and candidate optimization. However, computational predictions must remain anchored to experimental validation. Recent advances in natural-product discovery increasingly integrate computational and artificial-intelligence methods with structural, omics, and biological data, offering the possibility of accelerating dereplication, target identification, and lead prioritization [4]. The most informative workflows will be those in which in silico predictions generate experimentally testable hypotheses and experimental results iteratively refine the computational model.

A major challenge across the field remains the gap between experimental bioactivity and human efficacy. For many phytochemicals, poor aqueous solubility, chemical instability, intestinal transformation, extensive first-pass metabolism, rapid elimination, and limited tissue distribution reduce exposure to the parent compound ([1], Contributions 2 and 3). Consequently, concentrations commonly used in cell culture may exceed those achievable through dietary intake or conventional administration. Future mechanistic studies should therefore justify experimental concentrations using pharmacokinetic or physiologically plausible exposure data whenever possible.

Importantly, the parent molecule may not be the only, or even the principal, biologically relevant species in humans. Conjugated metabolites and microbiota-derived catabolites can differ substantially from the ingested compound in concentration, tissue distribution, and biological activity. Human studies demonstrate considerable interindividual variability in phenolic metabolism and bioavailability, with gut microbiota composition and activity emerging as major determinants alongside genetic and physiological factors [2]. This variability provides a strong rationale for incorporating metabolite profiling, microbiome analysis, and metabotype stratification into future intervention studies.

This perspective also changes how delivery technologies should be evaluated. Nanoformulations, encapsulation systems, prodrugs, and other delivery strategies should not be judged only by improved solubility or apparent cellular uptake. Their translational value depends on whether they reproducibly improve exposure at the relevant biological site, preserve or enhance target engagement, avoid new toxicological liabilities, and ultimately improve efficacy. Delivery technology should therefore be integrated with pharmacokinetic/pharmacodynamic analysis rather than considered an independent endpoint ([1], Contribution 3).

Complex mixtures introduce an additional level of uncertainty. Botanical extracts and food matrices may contain dozens or hundreds of compounds whose abundance and stability depend on biological and technological variables. Reproducibility requires detailed botanical authentication, extraction parameters, chromatographic fingerprints, quantitative marker compounds, contaminant control, and stability data. Where biological effects are attributed to a complex preparation, bioactivity-guided fractionation, chemical–biological correlation, or carefully designed mixture experiments can help identify the constituents or combinations responsible for the observed response.

## 3. Future Perspectives: Toward Mechanism-Based and Precision Bioactives

The next phase of bioactive-compound research should be driven by translationally informed experimental design. Chemical characterization, mechanism, exposure, efficacy, and safety should be considered as interconnected components from the beginning of a study. This requires standardized materials, appropriate positive and negative controls, concentration–response relationships, orthogonal validation of molecular targets, and models that reproduce relevant physiological barriers and disease contexts. Human-relevant three-dimensional cultures, organoids, organ-on-chip systems, and well-characterized animal models may improve translational fidelity when used for clearly defined questions.

Precision approaches are also likely to become increasingly important. The substantial interindividual variability observed in polyphenol metabolism suggests that identical intake does not imply identical biological exposure [2]. Genetics, microbiome composition, metabolic phenotype, diet, age, sex, disease status, and concomitant medication can all modify response. Rather than treating this heterogeneity only as statistical noise, future trials can use it to identify responder phenotypes, metabotypes, and biomarkers that support more targeted nutritional or therapeutic strategies.

Sustainability should likewise be integrated with biological efficacy. The valorization of fruit by-products described by Nunes et al. [Contribution 1] illustrates how bioactive-compound research can connect health innovation with circular use of biological resources. Yet sustainability claims should be supported by extraction yield, solvent and energy demand, scalability, batch reproducibility, waste generation, and life-cycle considerations. A compound recovered from waste is not automatically sustainable if its isolation requires resource-intensive processing. Green extraction and process intensification should therefore develop alongside pharmacological characterization.

Finally, the field would benefit from clearer evidence hierarchies. Chemical presence does not demonstrate bioavailability; antioxidant capacity in a chemical assay does not establish intracellular antioxidant action; docking does not establish target binding; target modulation does not necessarily establish disease modification; and preclinical efficacy does not establish clinical benefit. Making these distinctions explicit will strengthen, rather than diminish, the value of bioactive-compound research by directing experiments toward the evidence needed at each stage of translation.

## 4. Conclusions

The seven papers assembled in the Special Issue “New Advances in Bioactive Compounds in Health and Disease” demonstrate the breadth of contemporary bioactive-compound research, from phenolic acids recovered from agri-food by-products and established molecules such as caffeic acid and hesperidin to bioactive combinations, traditional herbal formulations, soybean-derived proteins, and rationally designed derivatives [Contributions 1–7]. Collectively, they show that the field is moving from broad descriptions of biological activity toward molecularly resolved and increasingly integrative approaches.

The central scientific challenge is now to connect chemical identity with biologically relevant exposure, verified molecular mechanisms, reproducible phenotypes, and meaningful human outcomes. Addressing this challenge will require integration of advanced analytical chemistry, multi-omics, computational science, pharmacokinetics, formulation technology, microbiome research, physiologically relevant models, and carefully designed clinical studies [1,2,3,4]. Within such a framework, bioactive compounds can be evaluated not merely as promising natural molecules but as mechanistically defined interventions whose efficacy, safety, variability, and translational potential can be tested rigorously. The contributions to this Special Issue provide complementary examples of this progression and underscore the continuing potential of bioactive compounds to inform new strategies for health promotion, disease prevention, and therapeutic innovation.

## Data Availability

No new data were created or analyzed in this study. Data sharing is not applicable to this article.
